# Epithelial Membrane Protein 2 Suppresses Non-Small Cell Lung Cancer Cell Growth by Inhibition of MAPK Pathway

**DOI:** 10.3390/ijms22062944

**Published:** 2021-03-14

**Authors:** Yunxia Ma, Desiree Charlotte Schröder, Miljana Nenkov, Maryam Noor Rizwan, Mohamed Abubrig, Jürgen Sonnemann, José M. Murrieta-Coxca, Diana M. Morales-Prieto, Martin Westermann, Nikolaus Gaßler, Yuan Chen

**Affiliations:** 1 Section Pathology of the Institute of Forensic Medicine, Jena University Hospital, Friedrich Schiller University Jena, Am Klinikum 1, 07747 Jena, Germany; Yunxia.ma@med.uni-jena.de (Y.M.); desiree.schroeder97@gmail.com (D.C.S.); miljana.nenkov@med.uni-jena.de (M.N.); maryamnoor95@gmail.com (M.N.R.); Mohamed.abubrig@med.uni-jena.de (M.A.); Nikolaus.gassler@med.uni-jena.de (N.G.); 2 Department of Pediatric Hematology and Oncology, Children’s Clinic, Jena University Hospital, Am Klinikum 1, 07747 Jena, Germany; juergen.sonnemann@med.uni-jena.de; 3 Placenta-Labor, Jena University Hospital, Am Klinikum 1, 07747 Jena, Germany; josemartin.murrietacoxca@uni-jena.de (J.M.M.-C.); Diana.Morales@med.uni-jena.de (D.M.M.-P.); 4 Electron Microscopy Center, Jena University Hospital, Ziegelmühlenweg 1, 07743 Jena, Germany; martin.westermann@med.uni-jena.de

**Keywords:** NSCLC, epithelial membrane protein, tumor pathways, exosome, microRNA

## Abstract

Epithelial membrane proteins (EMP1-3) are involved in epithelial differentiation and carcinogenesis. Dysregulated expression of EMP2 was observed in various cancers, but its role in human lung cancer is not yet clarified. In this study, we analyzed the expression of EMP1-3 and investigated the biological function of EMP2 in non-small cell lung cancer (NSCLC). The results showed that lower expression of EMP1 was significantly correlated with tumor size in primary lung tumors (*p* = 0.004). Overexpression of EMP2 suppressed tumor cell growth, migration, and invasion, resulting in a G1 cell cycle arrest, with knockdown of EMP2 leading to enhanced cell migration, related to MAPK pathway alterations and disruption of cell cycle regulatory genes. Exosomes isolated from transfected cells were taken up by tumor cells, carrying EMP2-downregulated microRNAs (miRNAs) which participated in regulation of the tumor microenvironment. Our data suggest that decreased EMP1 expression is significantly related to increased tumor size in NSCLC. EMP2 suppresses NSCLC cell growth mainly by inhibiting the MAPK pathway. EMP2 might further affect the tumor microenvironment by regulating tumor microenvironment-associated miRNAs.

## 1. Introduction

Lung cancer is one of the most genetically complex, aggressive, and lethal solid malignancies [1]. About 228,820 new cases of lung cancer (116,300 in men and 112,520 in women) are estimated for 2020 throughout the USA [2], and it is the leading cause of cancer-related death worldwide. Histologically, lung cancer is categorized into two main types, namely, small cell lung carcinoma (SCLC, 15% of all lung cancers) and non-small cell lung cancer (NSCLC, 85% of all lung cancers). NSCLCs can be further subclassified into several groups, containing squamous cell carcinoma (SCC), adenocarcinoma (ADC), and large cell carcinoma (LCLC) [3]. Over the past two decades, progress has been made in management of lung cancer due to early diagnosis through screening with low-dose computed tomography and implementation of biomarker-driven therapy, including targeted therapy and immunotherapy; however, the five-year survival rate is still lower than 21% [4]. As lung cancer is a heterogeneous disease, multiple predictive and prognostic biomarkers need to be identified to ensure better patient response to treatment. Characterization of novel biomarkers could help to further understand the molecular biology of the disease, improve early diagnosis, and develop new personalized therapies, which may eventually contribute to better clinical outcomes.

In previous studies, we characterized the tumor suppressor gene HOPX which exerted the tumor growth inhibitory function via RAS-induced senescence [5,6,7]. In order to identify genes potentially regulated by HOPX, we performed cDNA microarray analysis to compare the gene expression between HOPX transfectants and mock cells. The epithelial membrane protein 2 (EMP2) was one of the genes found to be upregulated in HOPX-transfected cells with more than 4-fold changes compared to mock cells (Table S1).

The epithelial membrane proteins (EMP1-3) belong to the peripheral myelin protein 22-kDa (PMP22) gene family containing at least seven members: PMP22, EMP1, EMP2, EMP3, PERP, brain cell membrane protein 1, and MP20 [8,9]. EMP1 is an integral membrane glycoprotein and is detectable in epithelial tissue from the gastrointestinal tract, skin, lungs, and brain [10]. A study from Taylor et al. depicted that EMP2 and EMP3 were expressed in most human tissue, with an expression pattern partially similar to that of EMP1 [11]. EMP1-3 alterations have been reported in different cancer types. In NSCLC, EMP1 was associated with clinical resistance of gefitinib [12], EMP2 gene silencing resulted in activation of ERK and JNK in NSCLC cells [13], and EMP3 expression was significantly lower in tumor tissues compared to healthy lung tissues [14].

Exosomes are small lipid bilayer membrane vesicles (30–150 nm), containing a variety of active molecules including proteins, mRNA, microRNA, and long noncoding RNA [15]. Accumulating evidence showed that exosomal miRNAs play a critical role in lung cancer progression and can be used as potential biomarkers for lung cancer diagnosis, prognosis, and therapy [16]. For example, tumor-derived exosomal miR-1247-3p induces cancer-associated fibroblast activation to foster lung metastasis of liver cancer [17]. Tumor-derived exosomal miRNAs, including adenocarcinoma-specific miR-181-5p, miR-30a-3p, miR-30e-3p and miR-361-5p, as well as SCC-specific miR-10b-5p, miR-15b-5p, and miR-320b, are considered to be effective biomarkers for early lung cancer diagnosis [18].

Currently, the tumor biological role of EMP2 and its impact on miRNA expression, particularly exosomal miRNA expression, in NSCLC cells is not yet understood. In this study, we combined EMP2, exosomes, and miRNA into one research frame to investigate the functional role of EMP2, its influence on the expression of exosomal miRNAs, and the effects of EMP2-derived exosomes on tumor cell migratory behavior.

## 2. Results

### 2.1. Expression of EMP1-3 in NSCLC Cell Lines and Primary Lung Tumor Samples

**Figure 1:**
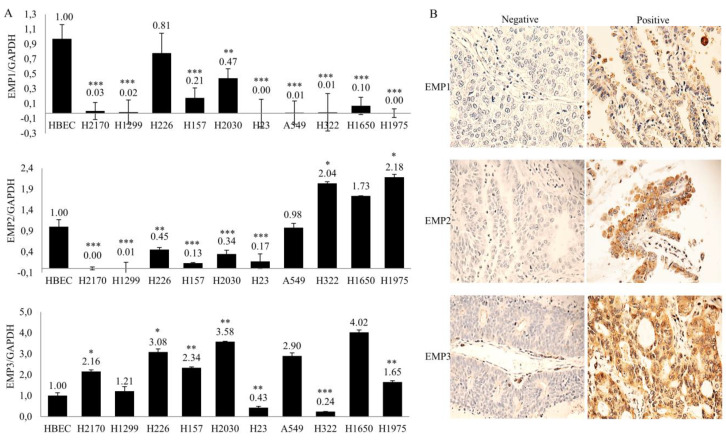
Expression analysis of epithelial membrane proteins (EMP) 1-3 in non-small cell lung cancer (NSCLC). (**A**) Analysis of EMP1-3 mRNA expression in 10 NSCLC cell lines by real-time RT-PCR. Gene expression in comparison to the housekeeping gene (GAPDH) expression in normal bronchial epithelial cells (HBECs) was set to 1.0. The data are shown as the means of three independent experiments ± standard deviation. * *p* < 0.05, ** *p* < 0.01, and *** *p* < 0.001 when analyzed with Student’s t-test. (**B**) Representative expression of EMP1-3 proteins in primary lung tissues (magnification ×400) using immunohistochemistry with negative staining (score 0; top) and strong staining (score 3; bottom).

We performed real-time RT-PCR to analyze EMP1-3 mRNA expression in ten NSCLC cell lines. Compared to human bronchial epithelial cells (HBEC), EMP1 was significantly downregulated in nine out of ten NSCLC cell lines and decreased expression of EMP2 was found in six out of ten cell lines, while EMP3 was upregulated in more than half (six out of ten) of NSCLC cell lines (Figure 1A).

In primary lung tumors, the protein expression of EMP1-3 was analyzed by immunohistochemistry on tissue microarrays (TMAs). Of the lung tumor samples, 52.7% (29 out of 55), 67.3% (37 out of 55), and 45.4% (25 out of 55) exhibited no EMP1, EMP2, and EMP3 expression, respectively. Lower expression of EMP1 was significantly related to tumor size (*p* = 0.004) (Table 1). The expression of EMP2 and EMP3 did not differ by gender, age, or tumor stages. The expression pattern for EMP1-3 was cytoplasmic. Representative images of EMP1-3 proteins expression are shown in Figure 1B. 

### 2.2. Evaluation of EMP1 and EMP2 DNA Methylation in NSCLC Cells

We selected NSCLC cell lines with decreased EMP1 and EMP2 mRNA levels for demethylation testing. After treatment with a DNA methyltransferase inhibitor, 5-aza-2′-deoxycytidine (DAC), EMP1 mRNA expression markedly increased in three NSCLC cell lines, including H2170, H23, and A549 (Figure S1), while EMP2 mRNA was enhanced in six NSCLC cell lines, namely, H2170, H1299, H226, H2030, A549, and H1650 (Figure S2).

Bisulfite sequencing (BS) was carried out to analyze the methylation status of EMP1 and EMP2 in the promoter region and exon 1. Partial DNA methylation of EMP1 was found only in the H23 cell line in the region from −33 to +220 bp containing nine CpG dinucleotides; no methylation was detected by BS in the EMP2 DNA (Table S2).

### 2.3. EMP2 Inhibits Tumor Cell Proliferation, Colony Formation, Migration, and Invasion

To determine the function of EMP2 in NSCLC cells, an expression vector containing the full-length cDNA of EMP2 was transfected into H1299 and H2170 in which no endogenous expression of EMP2 was found (Figure 1A).

**Figure 2:**
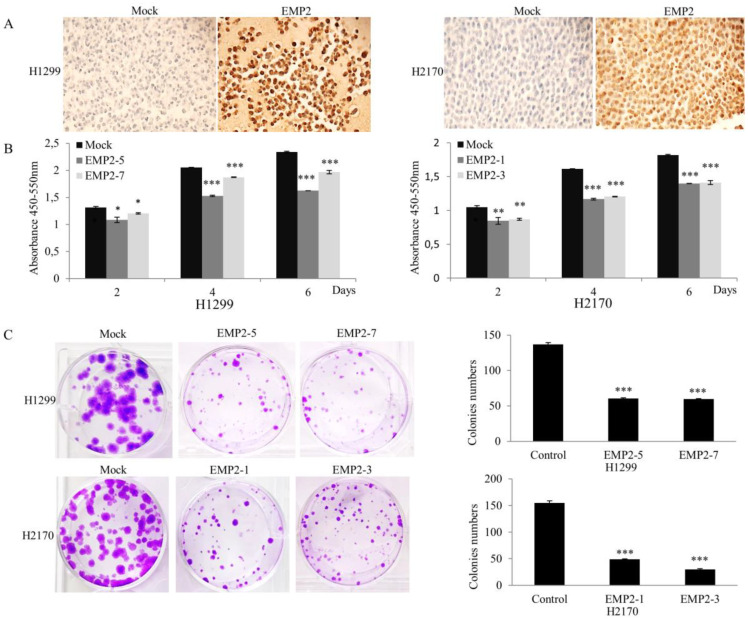
Influence of EMP2 overexpression on NSCLC cell growth. (**A**) EMP2 overexpression in H1299 and H2170 cell lines after stable transfection compared to mock cells was proven by cell-block analysis, confirming a successful transfection of EMP2 (magnification ×200) (**B**) EMP2-positive transfectant cells (EMP2-5 and EMP2-7 for H1299; EMP2-1 and EMP2-3 for H2170) showed significantly reduced cell proliferation compared to mock cells as revealed by BrdU cell proliferation assay. (**C**) Colony formation assay revealed that EMP2 overexpression resulted in significantly decreased colony formation in both H1299 and H2170 cells. The data presented are the means ± SE from three independent experiments. * *p* < 0.05, ** *p* < 0.01, and *** *p* < 0.001 when analyzed with Student’s *t*-test.

After stable transfection, overexpression of EMP2 in EMP2-5, EMP2-7, and EMP2-8 (H1299) transfectants, as well as EMP2-1, EMP2-2, and EMP2-3 (H2170), were confirmed by real-time RT-PCR (Figure S3), Western blotting (Figure S4), and immunohistochemistry on (Figure 2A).

As shown in Figure 2B and Figure S5, ectopic EMP2 expression significantly inhibited NSCLC cell proliferation in comparison to mock control on day 1, 3, and 5 (*p* < 0.05, *p* < 0.01, or *p* < 0.001).

A colony formation assay showed that EMP2 transfectants formed significantly reduced number of colonies in comparison to mock transfectants (*p* < 0.001; Figure 2C).

**Figure 3:**
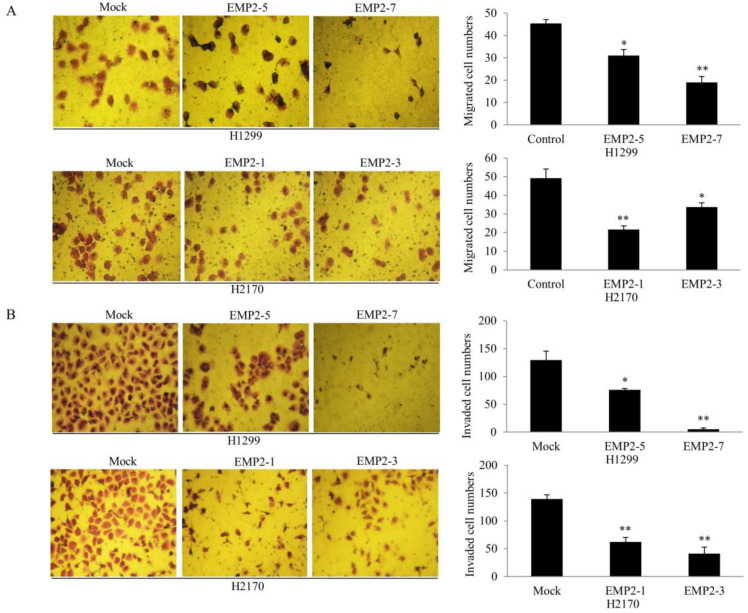
Effects of EMP2 overexpression on the migration and invasion of NSCLC cells. (**A**) Migration assay revealed that EMP2 suppressed migratory ability of the NSCLC cell lines H1299 and H2170 (40× objective). Quantification of cell migration (right). The data presented are the means ± SE from three independent experiments. (**B**) Invasion assay showed that EMP2 inhibited the invasive ability of NSCLC cells (40× objective). Quantification of invaded cells (right). The data presented are the means ± SE from three independent experiments. * *p* < 0.05 and ** *p* < 0.001 when analyzed with Student’s *t*-test.

A migration assay showed that the number of the migrated cells was significantly lower in EMP2-transfectants compared to mock cells (H1299: *p* < 0.05 or *p* < 0.01; H2170: *p* < 0.05, Figure 3A). Additionally, an invasion assay revealed that the number of invaded cells was markedly reduced in EMP2 transfectants compared with control cells (H1299: *p* < 0.05 or *p* < 0.01; H1299: *p* < 0.01; Figure 3B). These results suggested that EMP2 may inhibit both the migration and invasive ability of NSCLC cells.

### 2.4. Knockdown of EMP2 by siRNA Increases Cell Invasion Property

**Figure 4:**
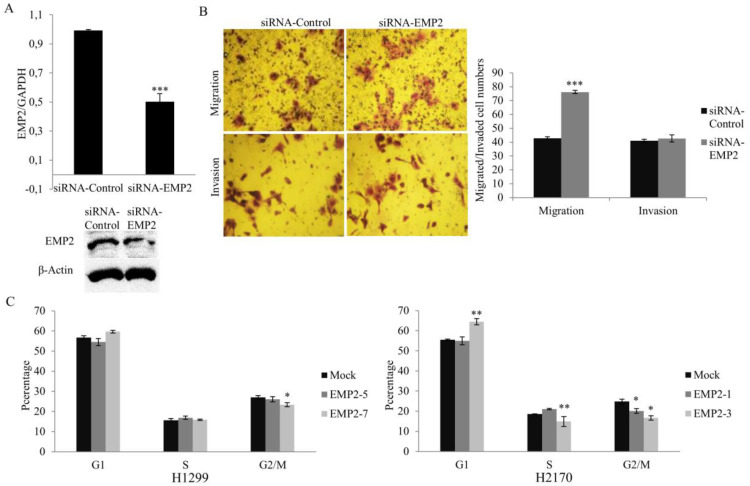
Effect of EMP2 knockdown on cell migration and invasion as well as the impact of EMP2 overexpression on the cell cycle. (**A**) Decreased expression of EMP2 in H1650 cells by siRNA knockdown was confirmed with real-time RT-PCR (top) and Western blotting (bottom). (**B**) Migration assay revealed that knockdown of EMP2 increased migratory ability of H1650 (50× objective; top), but the invasion assay did not reveal a significantly enhanced ability of tumor cell invasion after EMP2 siRNA knockdown (50× objective; bottom); quantification of migrated and invaded cell (right). Cells transfected with scrambled siRNA (siRNA Control) were used as control. (**C**) Flow cytometry showed that EMP2 overexpression led to a reduced H1299 cell population in G2/M, alongside an increased H2170 cell population in G1 and decreased cell populations in the S and G2/M phases. * *p* < 0.05, ** *p* < 0.01, *** *p* < 0.001 when analyzed using Student’s *t*-test.

We performed RNA interference to knockdown EMP2 in the cell line H1650, which exhibited high endogenous EMP2 expression on both the mRNA (Figure 1) and protein levels (Figure S6). Downregulation of EMP2 by siRNA was confirmed by real-time RT-PCR and Western blotting (Figure 4A). Contrary to ectopic expression, knockdown of EMP2 led to significantly increased migratory potential (*p* < 0.001), but not invasive ability, compared to control cells (Figure 4B).

### 2.5. Ectopic Expression of EMP2 Results in Alteration of Cell Cycle-Associated Genes/Proteins and the AKT and MAPK Pathways

To determine if EMP2 influences the cell cycle, we performed flow cytometric analysis of propidium iodide-stained nuclei. As shown in Figure 4C, in the cell line H1299, EMP2 overexpression resulted in a significantly reduced number of cells in the G2/M phase (*p* < 0.05). In the cell line H2170, overexpression of EMP2 led to a significantly increased number of cells in the G1 phase (*p* < 0.01), and a correspondingly reduced number of cells in the S (*p* < 0.05) and G2/M phases (*p* < 0.05). The data indicated that EMP2 might be associated with G1 cell cycle arrest in H2170 cells.

**Figure 5:**
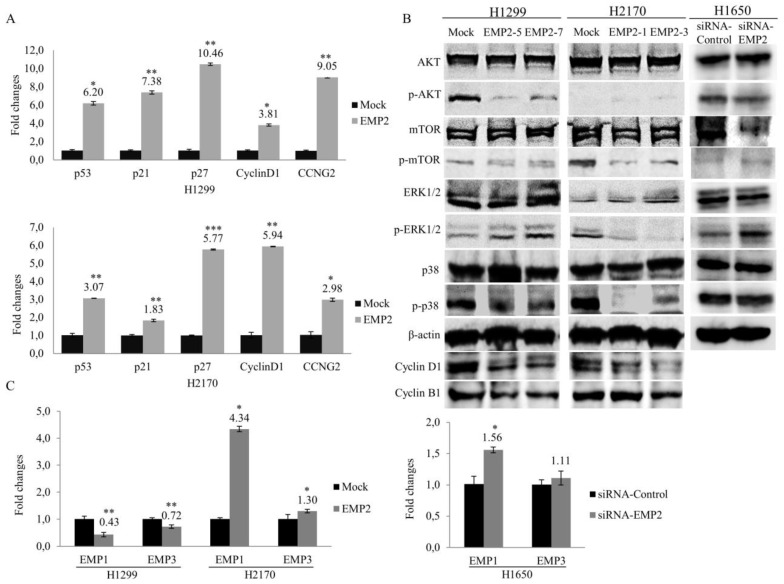
Influence of EMP2 on cell cycle regulatory genes/proteins and tumor-associated signal pathways. (**A**) Real-time RT-PCR analysis revealed altered expression of cell cycle regulatory genes in both H1299 and H2170 cells after EMP2 stable transfection. (**B**) Western blot analysis showed decreased phosphorylated levels of AKT and p38 as well as decreased protein expression levels of cell cycle regulators cyclin D1 and cyclin B1, and an increased phosphorylated level of ERK1/2 in H2199 cells. In H2170 cells, decreased phosphorylated levels of mTOR, ERK1/2, and p38 as well as decreased protein expression levels of cyclin D1 and cyclin B1 (clone EMP2-3) after EMP2 overexpression were found. An increased phosphorylated level of ERK1/2 after EMP2-knockdown was detected in H1650 cells (**C**) Influence of EMP2 overexpression (left) and knockdown (right) on EMP1 and EMP3 expression. * *p* < 0.05, ** *p* < 0.01, *** *p* < 0.001 when analyzed using Student’s *t*-test.

To investigate the molecular changes during cell cycle alteration, we evaluated the mRNA expression of the genes which are involved in the cell cycle regulation such as p53, p21, p27, cyclin D1, and CCNG2, showing that ectopic expression of EMP2 resulted in significant upregulation of all of these genes in both H1299 and H2170 (*p* < 0.05, *p* < 0.01, or *p* < 0.001; Figure 5A).

Cyclin D1 positively regulates the progression of the cell cycle through G1 phase. In line with G1 cell cycle arrest after EMP2 overexpression, we found decreased protein level of cyclin D1 in H1299 and H2170 cell lines (Figure 5B), indicating a post-transcriptional and/or post-translational regulation of cyclin D1 in the cells. Cyclin B1 is a key protein regulating cell cycle progression from G2 to M phase. The reduced cell population in G2/M phase after EMP2 overexpression might be partially explained by the decreased expression level of cycle B1 in both cell lines. However, the involvement of other cell cycle proteins cannot be ruled out and warrants further investigations. Nevertheless, downregulation of cyclin B1 and cyclin D1 accords with the decreased cell proliferation caused by EMP2 overexpression as revealed by BrdU assay (Figure 2B), WST assay (Figure S5), and colony formation assay (Figure 2C).

To examine the possible mechanisms by which EMP2 exerts cell growth inhibitory effects in NSCLC, Western blotting was carried out, showing that overexpression of EMP2 had different effects on alterations of pathways in H1299 and H2170 cells (Figure 5B). For the lung adenocarcinoma cell line H1299, reduced phosphorylated (p-) AKT and p-p38 were found in the EMP2 transfectants, while the expression level of p-ERK1/2 was enhanced compared to mock cells. In the lung squamous carcinoma cell line H2170, the expression levels of p-mTOR, p-ERK1/2, and p-p38 were reduced in EMP2-overexpression. The data indicated that EMP2 may influence signaling pathways in a cell type-dependent manner.

We also analyzed the expression levels of p-AKT, p-mTOR, p-ERK1/2, and p-p38 after EMP2-knockdown in H1650. The level of p-ERK1/2 was shown to be increased, while no obvious changes were found in the expression levels of p-AKT, p-mTOR, and p-p38 (Figure 5B). The densitometric quantification of the data from the Western blotting is shown in Supplementary Figure S7.

**Figure 6:**
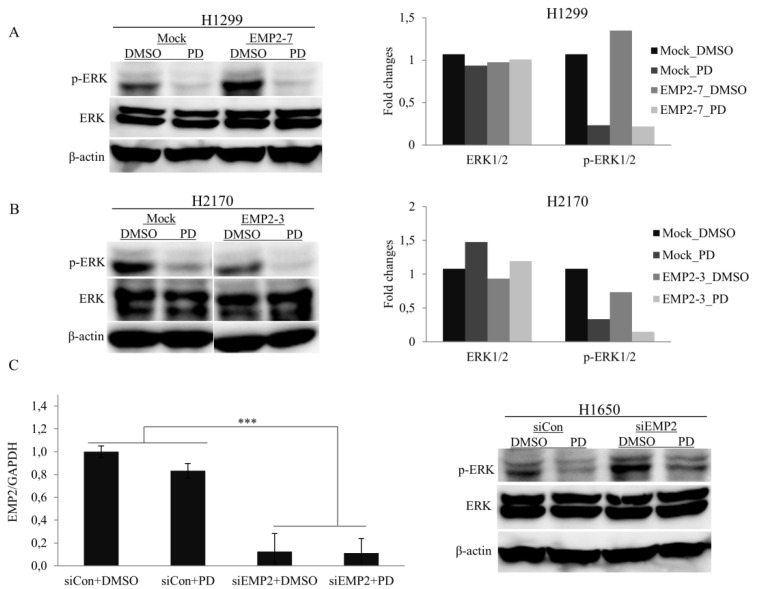
Influence of MEK inhibitor PD98059 (PD) on the expression level of phosphorylated ERK1/2 in NSCLC cells with ectopic expression of EMP2 or EMP2-knockdown. (**A**) PD98059 treatment led to a reduced expression level of p-ERK1/2 in H1299 cells. The expression level of p-ERK1/2 was enhanced in EMP2-transfected H1299 cells (EMP2-7) compared to mock cells after DMSO treatment. No altered expression level of p-ERK1/2 was found in EMP2-transfected H1299 cells compared to mock cells after PD98059 treatment (left). Densitometric quantification of the data from the Western blot analysis (right). (**B**) PD98059 treatment led to a reduced expression level of p-ERK1/2 in H2170 cells. In EMP2-transfected H2170 cells (EMP2-3), a decreased expression level of p-ERK1/2 was observed compared to mock cells after PD98059 treatment (left). Densitometric quantification of the data from the Western blot analysis (right) (**C**) A successful knockdown of EMP2 was confirmed by real-time RT-PCR analysis in H1650 cells treated with DMSO or PD98059 (left). H1650 cells treated with PD98059 showed reduced expression of p-ERK1/2 compared to cells treated with DMSO. Cells with EMP2 knockdown (siEMP2) exhibited more expression of p-ERK1/2 compared to mock cells (siCon) after PD98059 treatment (right). *** *p* < 0.001 when analyzed using Student’s *t*-test.

To further confirm the effects of EMP2 on the MAPK pathway, we treated the cells with a mitogen activated protein kinase kinase (MEK) inhibitor PD098059. PD98059 treatment generally led to decreased expression levels of p-ERK1/2 in EMP2 transfectants, cells with EMP2-knockdown, and mock control cells compared to DMSO treatment (Figure 6). In line with the data shown in Figure 5B, an increased level of p-ERK1/2 was observed in EMP2-transfected H1299 cells with DMSO treatment, and the expression level of p-ERK1/2 in EMP2-transfected cells did not differ from that in mock cells after PD98059 treatment (Figure 6A). In EMP2-transfected H2170 cells, a reduced expression level of p-ERK1/2 was observed compared to mock cells after PD98059 treatment (Figure 6B). Compared to mock cells, H1650-siEMP2 cells still showed more expression of p-ERK1/2 after PD98059 treatment (Figure 6C). The data from drug treatment suggest that EMP2 overexpression is specifically associated with reduced MAPK activity. Based on our data, considering that both p-p38 and p-ERK1/2 are two key components of the MAPK pathway, we conclude that EMP2 suppressed tumor cell growth mainly by inhibition of MAPK pathway in NSCLC.

Additionally, altered EMP2 expression was observed to affect EMP1 and EMP3 expression levels. As shown in Figure 5C, the mRNA levels of EMP1 and EMP3 were significantly downregulated in H1299 but upregulated in H2170. This observation, together with the Western blot analysis data showing different altered patterns of key molecular players in the AKT and MAPK pathways in two transfected cell lines, suggested that different molecular mechanisms might be involved in the EMP2-mediated growth inhibitory behaviors in lung ADC and SCC cells.

### 2.6. Characterization of Exosomes Secreted by NSCLC Cell Line H2170

**Figure 7:**
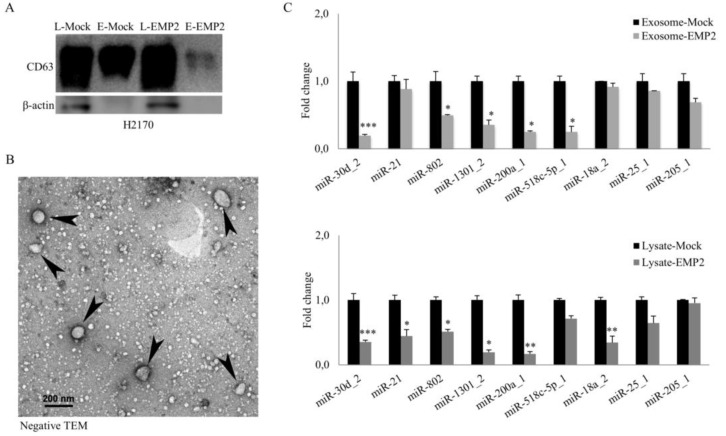
Characterization of exosomes secreted by H2170-transfected cells and altered miRNA expression in EMP2 overexpression. (**A**) Western blot analysis of the exosome marker CD63 from both cell lysates and exosomes. (**B**) The presence of membranous vesicles in the diameter range of 40–100 nm (arrowheads), as confirmed by transmission electron microscopy (TEM) analysis. (**C**) Overexpression of EMP2 significantly altered the expression of miRNAs related to regulation of the tumor microenvironment. * *p* < 0.05, ** *p* < 0.01, *** *p* < 0.001 when analyzed using Student’s *t*-test.

To characterize exosomes isolated from both EMP2-transfected cells and mock cells, Western blotting was performed. As shown in Figure 7A, expression of the exosome marker CD63 was detected in the isolated exosomes and was also present in the cell lysates. This could be explained by the fact that lung tissues exhibit endogenous CD63 expression (https://www.proteinatlas.org/ENSG00000135404-CD63/tissue) (accessed on: 13 March 2021). Additionally, transmission electron microscopy (TEM) analysis confirmed the presence of membranous vesicles in the diameter range of 40–100 nm (Figure 7B).

### 2.7. Alterations in miRNA Expression in Both Exosomes and Cell Lysates in EMP2 Overexpression

Analysis of the expression of nine miRNAs (miR-30d_2, miR-21, miR-802, miR-1301_2, miR-200a_1, miR-518-5p_1, miR-18a_2, miR-25_1, and miR-205_1) involved in cancer development in both exosomes and cell lysates from the EMP2 transfectants and mock cells was carried out. As shown in Figure 7C, the expression of exosome miRNAs, including miR-30d_2, miR-802 and miR-1301_2, miR-200a_1, and miR-518c-5p_1, was significantly decreased in EMP2-transfected H2170 cells compared to the mock control. For the miRNA isolated from the cell lysates, miR-30d_2, miR-21, miR-802, miR-1301_2, miR-200a_1, and miR-18a_2 expression was significantly downregulated in EMP2 overexpression. 

### 2.8. Uptake of Exosomes by H2170 Cells

**Figure 8:**
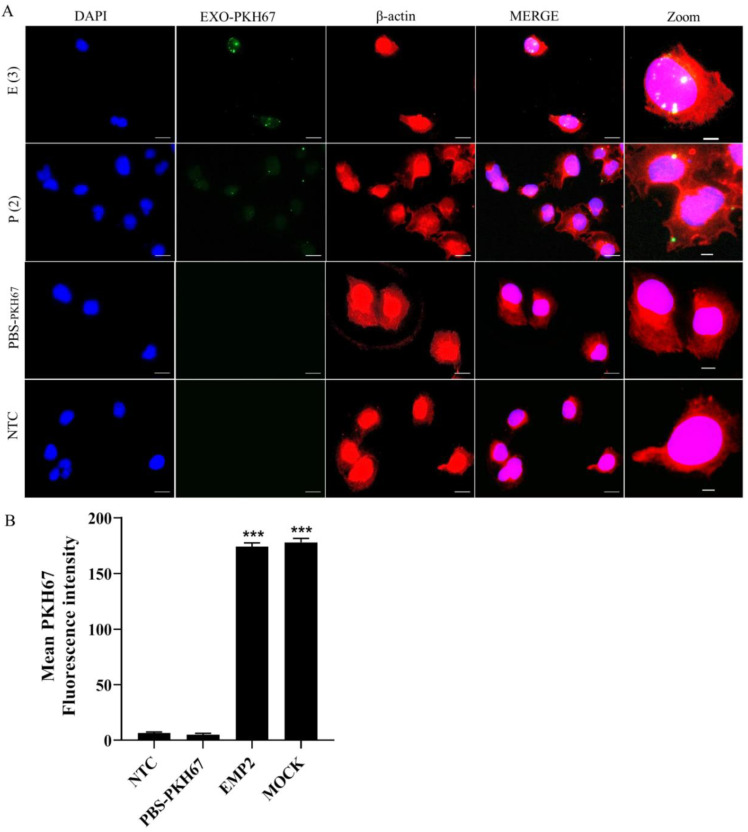
Uptake of exosomes by autologous cells. (**A**) Cellular uptake of autologous exosomes in H2170 cells imaged by confocal microscopy. The fluorescence of DAPI, β-actin, and that of EXOSOME-PKH67 are labeled with blue, red, and green, respectively. Scale bars: 10 µm. (**B**) Summary bar graphs of the relative mean fluorescence intensity (MFI) of PKH67 (exosomes) in recipient cells. The results are illustrated as the mean ± SEM of the relative MFI. *** *p* < 0.001 in comparison with nontreated cells (NTC) analyzed by one-way ANOVA with Bonferroni’s multiple comparisons test; E-Mock: Exosome of mock cells; E-EMP2: Exosomes of EMP2-transfected cells.

As shown above, EMP2 overexpression affected the composition of exosomal miRNA. Therefore, to determine if exosomes could be taken up by H2170 cells and if the uptake of exosomes could influence tumor migratory ability, uptake of PKH67-stained vesicles was visualized by confocal microscopy in H2170 cells incubated with EMP2PKH67 (exosome isolated from EMP2 transfectant stained with PKH67), MOCKPKH67 (exosome isolated from mock cells stained with PKH67), PBSPKH67 (PBS stained with PKH67), or a negative control (NTC) which did not contain PKH67 or exosomes. Figure 8A shows a merged image indicating that cellular uptake of exosomes could only be found in cells treated with EMP2PKH67 or MOCKPKH67, but not in PBSPKH67 or in NTC-treated cells. After measuring the mean PKH67 fluorescence intensity, we found no difference in the uptake of exosomes between cells treated with EMP2PKH67. In comparison with the PBSPKH67 and NTC-treated cells, significantly increased uptake of exosomes was found in cells incubated with exosomes (Figure 8B), indicating that the H2170 cells indeed took up exosomes.

However, no notable alterations in migratory ability were observed between cells treated with exosomes from EMP2 transfectants and mock cells (data not shown).

## 3. Discussion

Epithelial membrane proteins (EMPs) contain four putative transmembrane domain structures and are encoded by the growth arrest-specific 3 (GAS3)/peripheral myelin protein 22 kDa (PMP22) gene family [19]. Accumulating evidence showed that EMPs exhibit both prometastatic and antimetastatic functions, which are likely involved in the complex mechanisms of cancer progression [20]. In this study, we analyzed the expression of EMPs and explored the biological function of EMP2 as well as exosomes derived from EMP2-overexpressing cells in NSCLC development.

Expression analysis revealed a different mRNA expression pattern of EMPs with significant downregulation of EMP1 (nine out of ten cell lines) and EMP2 (six out of ten cell lines), but significant upregulation of EMP3 in more than half of the cancer cell lines compared to HBEC. During primary lung tumor sample analyses, we found that lower expression of EMP1 were significantly associated with increased tumor size. A study from Sun et al. depicted that overexpression of EMP1 in nasopharyngeal cancer cells led to markedly reduced migration and invasion [21]. In oral squamous cell carcinoma, however, high expression of EMP1 was related to an increased capability to metastasize to nodes [22]. In our study, we did not find a significant correlation between EMP2 expression and clinical outcome. However, data from TCGA showed that EMP2 overexpression was associated with favorable prognosis in patients with lung cancer (https://www.proteinatlas.org/ENSG00000213853-EMP2/pathology/lung+cancer#imid_3898442) (accessed on: 13 March 2021).

Epigenetic modifications, including DNA methylation, are closely associated with cancer initiation and progression [23]. In our study, methylation analysis showed partial methylation of EMP1 only in one NSCLC cell line, indicating that DNA methylation might not be a main regulatory mechanism responsible for gene silencing of EMP1 and EMP2 in NSCLC. Indeed, methylation of EMP1 and EMP2 in human cancer is rarely reported, although methylation of EMP3 was previously detected in NSCLC [13] and glioblastoma [24], suggesting different gene regulatory programs in EMPs.

Functionally, EMP family members play important roles in the control of cell growth [25], indicating their role in cancer development. Indeed, a growing body of evidence showed the participation of EMPs in cancer initiation and progression. For example, EMP1 promotes tumor metastasis by enhancing cell migration in prostate cancer [26], whereas EMP2 acts as an oncogenic protein in endometrial cancer cells, with high EMP2 related to increased lymphovascular invasion and poor survival [27,28,29]. EMP3 is induced by TWIST1/2 and regulates epithelial-to-mesenchymal transition of gastric cancer cells [30]. In line with the role of EMP2 in nasopharyngeal cancer and melanoma [31,32], EMP2 overexpression inhibited cell proliferation, migration, and invasion and caused G1 cell cycle arrest, while downregulation of EMP2 by siRNA resulted in an enhanced cell migratory ability. We did not perform proliferation assay after EMP2 knockdown, since the downregulation of EMP2 by siRNA was transient and could be recovered after 3–4 days, while the observation time for the proliferation assay was 5-day long. Nevertheless, our data suggest that EMP2 may exert tumor growth inhibitory effects in human NSCLC.

The diverse functions of EMPs in human cancer are mediated by numerous signaling pathways, with abnormal activation of these signal pathways shown to influence tumor biological behaviors [33]. EMP2 promotes endometrial tumor formation and migration through activation of the FAK/Src pathway [27]. In urothelial carcinoma of the upper urinary tract, EMP2 interacts with integrins αV and β3 to regulate cell adhesion and migration [34]. In this study, ectopic expression of EMP2 resulted in decreased activity of AKT and increased activity of ERK in the lung ADC cell line H1299, while a slightly enhanced p-AKT level and reduced p-ERK1/2 level were observed in the SCC cell line H2170. This opposite altered protein expression pattern in the AKT and MAPK pathways was related to the different genetic backgrounds of the cells. Nevertheless, reduced p-p38, an important player in the MAPK pathway, was found in both cell lines after EMP2 overexpression. Furthermore, treatment with MEK inhibitor PD098059 led to lower expression of phospho-ERK1/2 in EMP2-transfected H2170 cells compared to mock-transfected cells. Lee et al. depicted that EMP2 gene silencing reduces the PP2A via ubiquitination induced by cav-1, which sequestered alpha4, leading to the activation of ERK and JNK in NSCLC cells [13]. In line with this observation, we detected an enhanced level of p-ERK1/2 after EMP2 knockdown. Moreover, upon PD098059 treatment, the expression level of p-ERK1/2 was still higher in cells with EMP2-knockdown compared to control cells. These data together further indicated an essential role of the MAPK signaling in EMP2-mediated inhibition of NSCLC cell growth. Since it is well recognized that aberrant activation of the MAPK pathway promotes cell proliferation, metastasis, and cell survival, we speculate that EMP2 might suppress NSCLC cell growth mainly by inhibiting the MAPK pathway. Additionally, the alteration of cell cycle regulators such as cycle D1 and cycle B1 might also contribute to the inhibited cell growth. Currently we do not exactly know how cyclin D1 could be regulated by EMP2 in NSCLC cells. Many studies demonstrated the post-transcriptional and post-translational regulation of cyclin D1 in different cellular contexts. Witzel et al. thought that different pathways might play a role in regulation of cyclin D1 post-transcription [35]. Guo et al. found that a change of protein stability might be involved in the post-translational induction of the cyclin D1 protein [36]. On the post-translational level, EMP2 might regulate cyclin D1 by influencing the protein stability of cyclin D1 directly or indirectly. Moreover, altered EMP1 and EMP3 mRNA levels in EMP2-transfected cells indicated that other molecular mechanisms might also be responsible for the growth inhibitory effects of EMP2. The precise mechanism of post-translational regulation of cyclin D1 by EMP2 and the interaction between epithelial membrane proteins in NSCLC should be further investigated in future studies.

The association between EMP2 and miRNAs in the development of human cancer, particularly in lung cancer, is not yet well elucidated. Recently, analysis of exosomal miRNAs, one of the important exosome cargos, received much attention since they deliver essential information in terms of cell-cell communication [37]. We selected nine miRNAs involved in carcinogenesis and analyzed their expression levels in both exosomes and cell lysates after stable transfection of EMP2. The expression patterns of miRNA in both cases exhibited similar tendencies upon EMP2 overexpression, with miR-30d_2, miR-802, miR-1301_2, and miR-200a_1 shown to be significantly downregulated in EMP2-transfected cells compared with mock control cells. However, significantly reduced levels of miR-21 and miR-18a_2 were only found in cells but not in the exosomes. This discrepancy might be explained by the fact that loading of miRNAs into exosomes is dependent on the presence of exosome motifs and the 3′-end characteristics of miRNA sequences [38]. These features regulate intracellular pathways, leading to different enrichment of singular miRNAs within exosomes, which might be differ from that in the original cells. With the exception of miR-1301_1, the roles of the other three miRNAs were extensively investigated in NSCLC, showing tumor suppressive functions. For instance, decreased expression of miR-30d_5p is frequently observed in NSCLC, suppressing tumor cell migration/invasion by targeting NFIB [39,40,41], miR-802 inhibits the aggressive behaviors of NSCLC cells by directly targeting FGFR1 [42], and miR-200a suppresses migration and invasion and enhances the radiosensitivity of NSCLC cells by inhibiting the HGF/c-Met signaling pathway [43]. Exosomal microRNAs emerged as key players in modulating the tumor microenvironment [44,45]. The influence of miR-30d, miR-802, miR-1301, and miR-200a regulating cell–cell communication in the tumor microenvironment was previously observed [44,46,47]. In a translational context, the clinical application of these miRNAs as crucial agents in targeted therapies should be further investigated.

Exosomes isolated from oncogenic protein-overexpressing cancer cells were found to be able to promote cell migration [48,49]. In our study, treatment with exosomes isolated from tumor cells transfected with EMP2 did not affect tumor cell migratory behavior, although the exosomes were shown to be successfully taken up by the cells. Given the role of EMP2 in regulation of miRNAs involved in modeling the tumor microenvironment, it might be interesting to explore the impact of exosomes from EMP2-overexpressing cells on paracrine signaling and the behavior of cancer—associated fibroblasts in future studies. 

Limitations of the study remain. On the one hand, the sample size of the primary lung tumors used for the EMP1-3 protein expression analysis was small, and on the other hand, the function of EMP2 was not assessed in animal models. Additionally, only a handful of tumor pathways were investigated, given the functional diversity of EMP2 in human cancers [33]. These drawbacks should be overcome by increasing sample size, performing in vivo analysis, and investigating mores signaling molecules in future studies. The observation that anti-EMP2 IgG1 slowed tumor growth in breast cancer xenografts without detectable systemic toxicity indicates its immunotherapeutic value for the treatment of tumors with positive EMP2 expression [50]. Further investigations are required to shed more light on the EMP-related functions and EMP-mediated signaling pathways in different types of cancer, which could be helpful in the development of EMP-targeted tumor therapies.

## 4. Materials and Methods

### 4.1. Cell Lines, Cell Cultures, Drug Treatments, and Cell-Block Preparation

Human bronchial epithelial cells (HBEC) were purchased from Clonetics (San Diego, CA, USA) and cultured in BEG media (Clonetics, San Diego, CA, USA). Ten human non-small cell lung cancer cell lines (H2170, H1299, H226, H157, H2030, H23, A549, H322, H1650, and H1975) were purchased from the American Type Culture Collection (ATCC, Rockville, MD, USA) and the German Collection of Microorganisms and Cell Culture (DSMZ, Braunschweig, Germany). The cells were cultured in RPMI 1640 medium containing 10% (*v*/*v*) fetal bovine serum (FBS) and 1% (*w*/*v*) glutamine, and maintained in a humidified atmosphere with 5% CO2 at 37 °C.

NSCLC cell lines were cultured in 6-well plates. Upon 50% confluence, the cells were treated with 5 μM of 5-aza-2′-deoxycytidine (DAC) (Sigma Chemical Co., St. Louis, MO, USA) on days 0, 2, and 4. Cells were then harvested for total RNA isolation. Before treatment with the MEK inhibitor PD98059, cells were serum-starved overnight. Afterwards, cells were incubated with 50 µM of PD98059 (Biozol, Eching, Germany) for 60 min.

For construction of the cell blocks, cell pellets were collected by centrifugation at 1000 g for 3 min as described previously [51].

### 4.2. RNA Extraction and Real-Time RT-PCR

Total RNA was extracted from cell lines and exosomes using Trizol reagent (PEQLAB, Erlangen, Germany) according to the manufacturer’s protocols. For gene expression analysis, reverse transcription with 500 ng of RNA was performed using a QuantiTect^®^ Reverse Transcription Kit (Qiagen, Hilden, Germany). For miRNA expression analysis, reverse transcription with 300 ng of RNA was carried out using a miScript II RT Kit (Qiagen, Hilden, Germany), according to the manufacturer’s instructions.

Real-time RT-PCR was performed in 0.1 mL tubes on the Rotor-Gene Q (Qiagen, Hilden, Germany) with the FastStart Universal SYBR Green Master (Roche AG, Mannheim, Germany). Twenty-five/fifteen nanograms of total RNA were used for mRNA/miRNA analysis. Glyceraldehyde-3-phosphate dehydrogenase (GAPDH) was used as an internal control for gene expression (mRNA) analysis. RNU6 was applied as an internal control for analysis of miRNA isolated from the cell lysates. For miRNA extracted from the exosomes, a cel-miR-39 Spike-In control (Norgen, Thorold, ON, Canada) was used as the internal control for normalization of the qRT-PCR data [52]. The 2^−ΔΔCq^ method was used for quantification [53]. The data were derived from three independent experiments. The primer sequences are listed in Table S3. The primers for the miRNA analysis were purchased from Qiagen.

### 4.3. Genomic DNA Isolation, Bisulfite Treatment, and Bisulfite Sequencing

Genomic DNA isolation, bisulfite modification, and bisulfite sequencing (BS) were performed as described previously [54]. The primers used for BS are listed in Table S3.

### 4.4. Tissue Microarray (TMA) Construction and Immunohistochemistry

A total of 55 primary lung tumor specimens obtained from the University Hospital Jena from 1995 to 2001 were included for the construction of the tissue microarray. None of the patients received adjuvant radiotherapy or chemotherapy before surgery. The study was approved by the local ethical committee of University Hospital Jena (Nr: 3815-07/13).

The TMA was constructed using a manual tissue arrayer (Beecher Instruments, Woodland, WI, USA). Each tumor sample was represented in the TMA by two tissue cylinders of 0.6 mm diameter, as described previously [7]. Immunohistochemistry (IHC) was carried out to analyze the protein expression of EMP2 on the cell blocks and EMP1-3 in the TMAs. Briefly, sections were deparaffinized with xylene and gradually hydrated. Antigen retrieval was conducted by treatment in a pressure cooker for 6 min. The working concentrations of the EMP1-3 antibodies are listed in Table S4. Detection was performed according to the manufacturer’s instructions (LSABTM 2-kits, DAKO). Immunohistochemistry was scored semiquantitatively as negative (0% positively stained cells; score 0), weak (<10% positively stained cells; score 1), moderate (10–25% positively stained cells; score 2), or strong (>25% positively stained cells; score 3). For statistical evaluation, scores of 0 were considered negative, while scores of 1, 2, or 3 were considered positive.

### 4.5. Stable Transfection

A full-length human-EMP2 cDNA purchased from GenScript (Piscataway, NJ, USA) was transfected into an adenocarcinoma cell line H1299 and a squamous lung cancer cell line H2170 exhibiting no endogenous expression of EMP2, following the instructions of Lipofectamine^®^ 2000 DNA Transfection Reagent Protocol (Thermo Fisher, Hamburg, Germany). An empty vector pcDNA3.1 was transfected as a control. Stable transfection was carried out as described previously [6].

### 4.6. Cell Proliferation Assay, Colony Formation Assay, Cell Migration and Invasion Assay, and Cell Cycle Analysis

For the proliferation assay, cells (1 × 10^4^ cells /well) were seeded in 96-well plates and incubated for 1, 3, or 5 days at 37 °C. Cell proliferation was analyzed by using a BrdU Cell Proliferation ELISA Kit (Abcam, Berlin, Germany) and a Cell Proliferation Reagent WST-1 (Roche Diagnostics, Mannheim, Germany) according to the manufacturers’ instructions. The absorbance at 450/550 (BrdU) or 450/690 nm (WST-1) was measured by using the microplate reader Tecan Infinite 200 PRO (Tecan, Maennedorf, Switzerland) for each well. The experiment was carried out in triplicate.

For the colony formation assay, cells (4000 cell/well) were seeded in 6-well plates. After 8–11 days, colonies were stained with crystal violet and colony numbers were counted.

For the migration assay, cells (3 × 10^4^ cells /well) were resuspended in 500 µL of RPMI1640 medium and placed in the upper transwell chamber (8 µm pore size, BD Biosciences). The upper chambers were placed in a 24-well culture dish containing 700 µL of medium with 10% (*v*/*v*) FBS. Cells were incubated at 37 °C for 24 h, and nonmigratory cells on upper chamber were removed using a moistening cotton swab. The migratory cells were then fixed with ice-cold methanol and stained with 0.5% crystal violet for 10 min at 37 °C. For the invasion assay, cells (1 × 10^5^ cells /well) were harvested after starvation for 24 h using FBS-free medium and seeded in matrigel-coated transwell chambers (BD Biosciences, Franklin Lakes, NJ, USA). Cells were incubated at 37 °C for 24 h. After removing uninvaded cells, the invaded cells were stained for calculation.

For analysis of the cell cylcle, cells (1.5 × 10^5^ cells /well) were seeded in 6-well plates and incubated for 72 h at 37 °C. Cell pellets were collected by centrifugation at 1000 rpm for 5 min, fixed in 2 mL of ice-cold 70% ethanol, and stored at −20 °C overnight. After washing, cells were resuspended in PBS containing 1% glucose, 50 μg/mL RNase A (Roche, Mannheim, Germany), and 50 μg/mL propidium iodide, followed by incubation for 45 min at 4 °C in the dark. Cell cycle distribution was analyzed using a BD FACSCanto™ II (BD Biosciences, Heidelberg, Germany). All experiments were performed in triplicate.

### 4.7. RNA Interference

H1650 cells with endogenous expression of EMP2 were seeded in a 6-well plate (3 × 10^5^ cells/well) and incubated overnight. The cells were transfected with EMP2 siRNA (sc-93419; Santa Cruz, Dallas, TX, USA) or scrambled siRNA (sc-37007; Santa Cruz, Dallas, TX, USA) using Lipofectamine 2000 (Thermo Fisher, Hamburg, Germany). EMP2 expression was assessed 48 h after transfection by real-time RT-PCR and Western blotting.

### 4.8. Western Blotting

Western blotting was performed as described previously [7]. Thirty-five micrograms of proteins were electrophoresed on 8% SDS-PAGE gels and blotted on a nitrocellulose membrane. Antibodies used for the Western blotting are listed in Table S4. Signals were visualized using an enhanced chemiluminescence detection system (Santa Cruz Biotechnology, Dallas, TX, USA) according to the manufacturer’s instructions. Mouse anti-beta-actin antibody was used as the loading control.

### 4.9. Exosome Isolation 

Cells were washed with PBS and cultured in RPMI 1640 medium supplemented with 5% (*v*/*v*) exosome-depleted FBS (ED-FBS) (Thermo Fisher, Hamburg, Germany). After 24 h, the cell media were collected and centrifuged at 2000× *g* for 30 min to remove cellular debris. Ultracentrifugation was performed as described by Théry et al. [55], with some modifications. Briefly, cell media were centrifuged at 19,000× *g* for 30 min, then the supernatants were filtrated through a filter (0.2–0.8 µm pore size), followed by centrifugation at 100,000× *g* for 70 min. Cell pellets were resuspended in PBS and subjected to centrifugation again at 100,000× *g* for 70 min. Exosomes were then dissolved in PBS for characterization using Western blot analysis and electron microscopy.

### 4.10. Negative Staining Transmission Electron Microscopy (TEM)

Carbon-coated copper grids (400 mesh, Quantifoil Micro Tools GmbH, Großlöbichau, Germany) were hydrophilized by glow discharge treatment at low pressure in air. Aliquots of 10 μL of the exosome solution were adsorbed onto hydrophilic grids for 20 s, washed twice with distilled water, then stained on a drop of 2% uranyl acetate. Samples were analyzed using a Zeiss EM902A electron microscope (Carl Zeiss AG, Oberkochen, Germany) operated at 80 kV accelerating voltage, and images were acquired with a sharp: eye 2 k × 2 k CCD-Camera (Tröndle TRS, Moorenweis, Germany).

### 4.11. Cellular Uptake of Exosomes Isolated from EMP2-Transfected H2170 Cells

Exosome uptake assay was carried out as previously described [56]. Exosomes derived from EMP2-transfected H2170 cells were labeled with 2 µM PKH67 dye (Sigma-Aldrich, Munich, Germany) for 5 min. The staining reaction was stopped by adding an equal volume of 1% ED-FBS in PBS. The cells were washed twice with PBS by centrifugation at 100,000× *g* for 70 min. H2170 cells were seeded into 6-well plates and incubated with PKH67-labeled exosomes (500 ng/mL) for 24 h in 2 mL RPMI 1640 medium containing ED-FBS. For confocal analysis, 7500 cells were seeded in Ibidi Chambered Coverslips (Ibidi GmbH, Gräfelfing, Germany) and treated with PKH67-stained exosomes or an equivalent volume of PBS/PKH67 as a control. After 24 h, cells were washed with ice-cold PBS and fixed with 2% formaldehyde for 10 min at RT. The slide was incubated with primary and secondary antibodies (Table S4) for 2 h and 60 min, respectively. DAPI (1 µg/mL; Sigma Aldrich) was added to visualize the nuclei. Cellular uptake of exosome was observed using a Zeiss LSM 710 confocal laser microscope (Carl Zeiss Microscopy GmbH) with 405 nm (DAPI) and 488 nm (PKH67) lasers. Confocal images and the mean fluorescence intensity (MFI) were analyzed with ZEN 2.3 imaging software (Carl Zeiss Microscopy GmbH).

### 4.12. Statistical Analysis

Statistical analysis was performed using the statistical software package SPSS21 (SPSS, Chicago, IL, USA). Two-tailed chi-square (χ^2^) and Fisher’s exact tests were performed to analyze the correlation between EMP1-3 protein expression and clinicopathological parameters. Student’s t-test was carried out to evaluate the differences between EMP2 transfectants and mock transfectant cells. One-way ANOVA with Bonferroni’s multiple comparisons test was performed to analyze exosomal uptake. Two sided *p* values were calculated, with *p* values < 0.05 considered to be statistically significant.

## Figures and Tables

**Table 1:** Correlation between EMP1 expression and clinicopathological data in primary lung tumor.

		EMP1		*p*-Value	
		0	1–3		
Type	ADC	13	12	0.146	Fisher’s
	SCC	15	9		
	Others	1	5		
Gender	Male	22	23	0.303	Fisher’s
	Female	7	3		
Age	≤65	20	17	1	
	>65	9	9		
pT	1–2	28	17	0.004	Fisher’s
	3–4	1	9		
pN	0–1	20	20	0.508	
	2–4	9	6		
pM	0	25	25	0.355	Fisher’s
	1–3	4	1		
Grade	1–2	18	11	0.143	
	3–4	11	15		

ADC, adenocarcinoma; SCC, squamous cell carcinoma. pT: tumor size; pN: tumor invasion in lymph node. pM: tumor metastasis

## Data Availability

Data is contained within the article or Supplementary Materials.

## Supplementary Materials

The following are available online at https://www.mdpi.com/1422-0067/22/6/2944/s1. Table S1: cDNA microarray data in comparison of HOPX transfectants to mock cells; Table S2: Methylation patterns of EMP1 and EMP2 in NSCLC cells; Table S3: Sequences of primers; Table S4: Antibodies used for Western blotting (WB) and immunohistochemistry (IHC), and immunofluorescence (IF); Figure S1: Reexpression of EMP1 after treatment with demethylation agent 5-aza-2′-deoxycytidine (DAC) in NSCLC cells; Figure S2: Reexpression of EMP2 after treatment with demethylation agent 5-aza-2′-deoxycytidine (DAC) in NSCLC cells; Figure S3: Overexpression of EMP2 mRNA after stable transfection with an EMP2 expression vector in NSCLC cell line H1299 (left) and H2170 (right); Figure S4: overexpression of EMP2 protein after stable transfection in H1299 and H2170 cells; Figure S5: Cell proliferation assay using WST-1 proliferation reagent. Figure S6: EMP2 protein expression NSCLC cell lines; Figure S7: Densitometric quantification of the data from Western blot analysis in Figure 5B.

## Abbreviation

EMP	epithelial membrane proteins
SCLC	small cell lung carcinoma
NSCLC	non-small cell lung carcinoma
ADC	adenocarcinoma
SCC	squamous cell carcinoma
PMP22	peripheral myelin protein 22-kDa
HBEC	human bronchial epithelial cells
ED-FBS	exosome depleted fetal bovine serum
DAC	5-aza-2′-deoxycytidine
GAPDH	glyceraldehyde-3-phosphate dehydrogenase
TMA	tissue microarray
TEM	transmission electron microscopy
MEK	mitogen activated protein kinase kinase
