# Supplementary figures and images for: Epithelial Membrane Protein 2 Suppresses Non-Small Cell Lung Cancer Cell Growth by Inhibition of MAPK Pathway
Source: Int J Mol Sci. 2021 Mar 14;22(6):2944. doi: 10.3390/ijms22062944 (PMC7999101; doi:10.3390/ijms22062944)

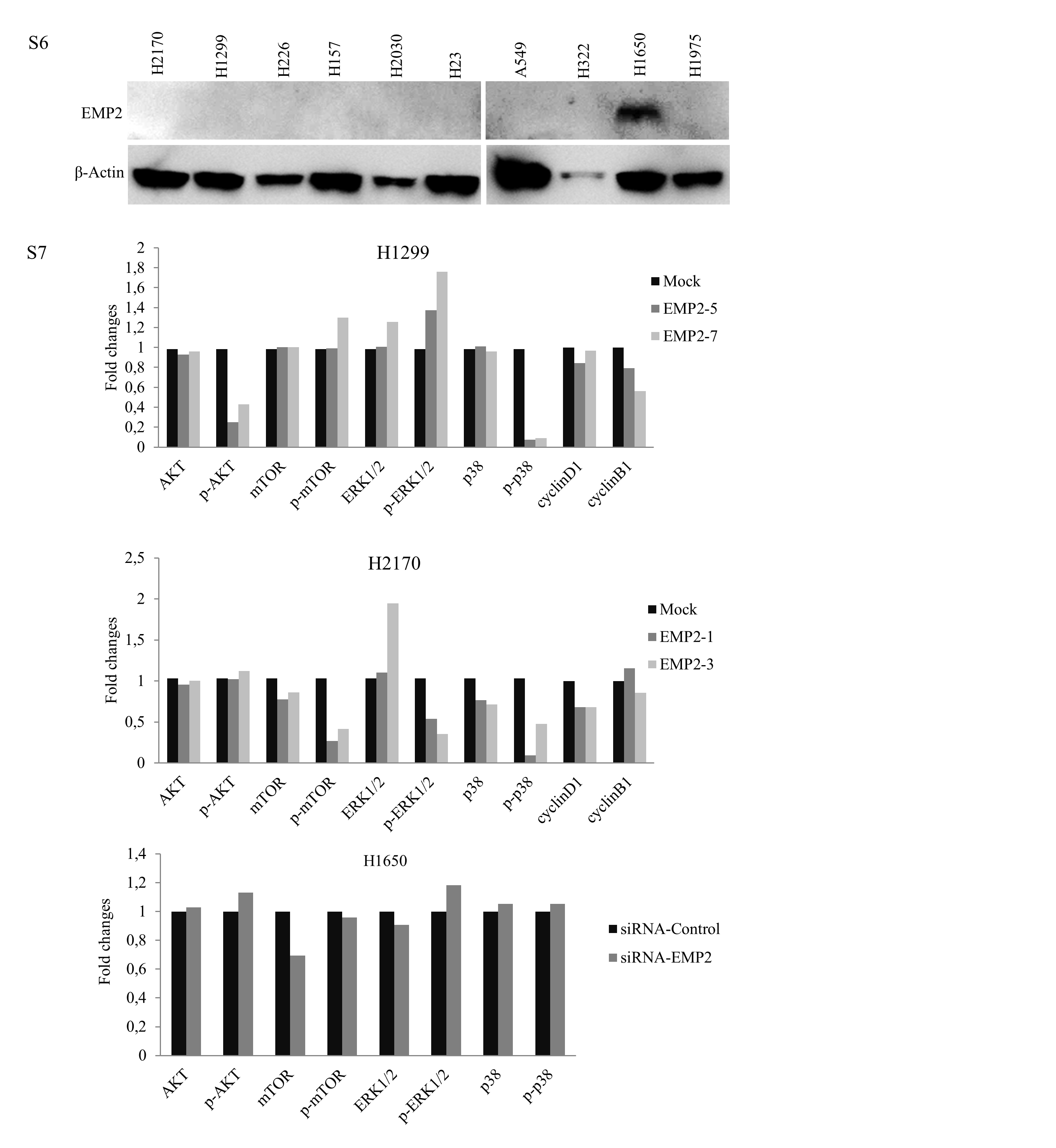

Supplement: Supplementary file 1 [file ijms-22-02944-s001.zip › Supplementary figure 2.TIF]

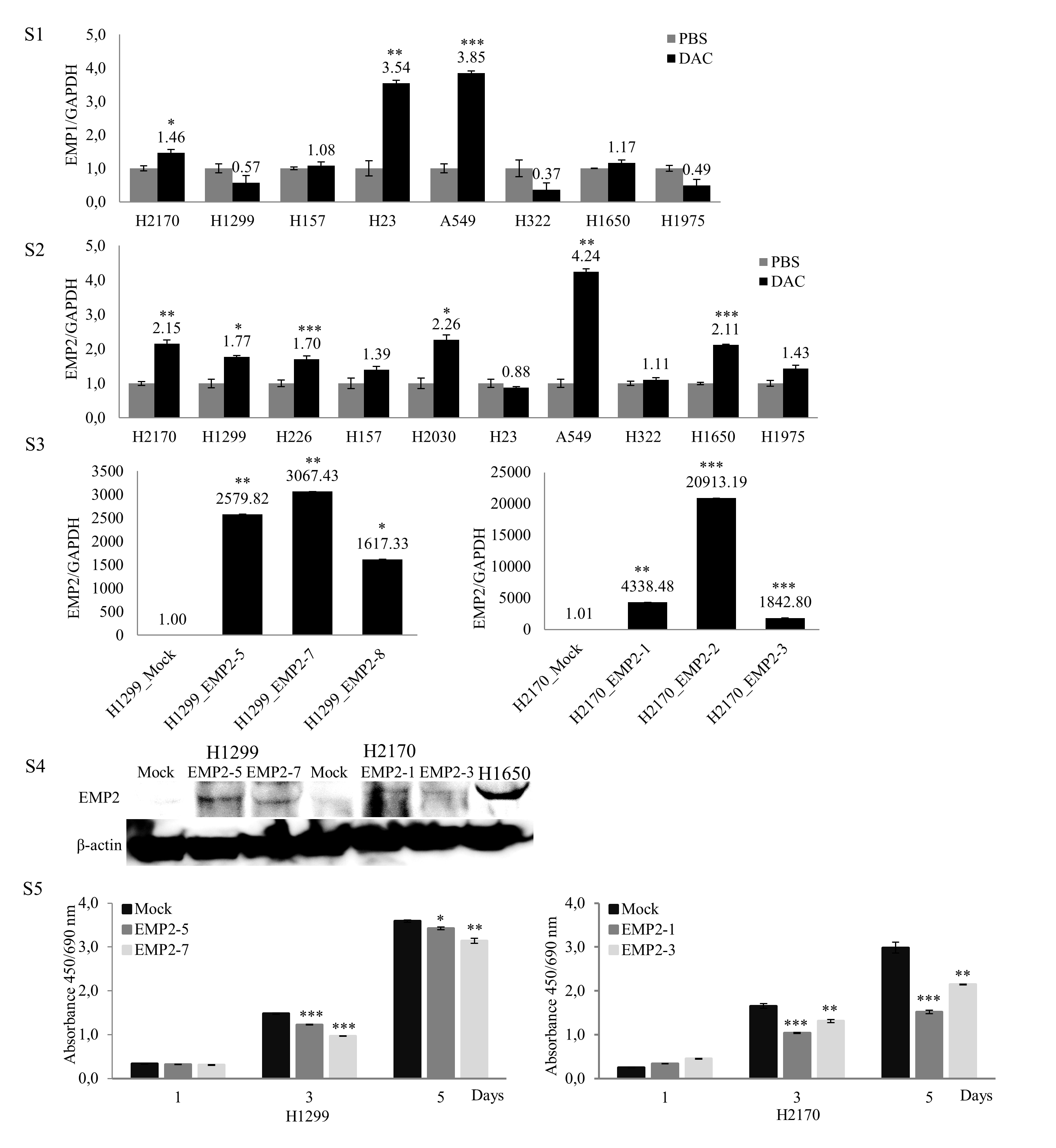

Supplement: Supplementary file 1 [file ijms-22-02944-s001.zip › Supplementary figure 1.TIF]
